# Health related quality of life of obese adolescents in Kuwait

**DOI:** 10.1186/1471-2431-13-105

**Published:** 2013-07-11

**Authors:** Shurooq A Boodai, John J Reilly

**Affiliations:** 1 University of Glasgow College of Medical, Veterinary, and Life Sciences, Yorkhill Hospitals, Glasgow G3 8SJ, Scotland; 2 Physical Activity & Public Health Science, Physical Activity for Health Group, School of Psychological Sciences & Health, University of Strathclyde, 40 George St, Glasgow G1 1XQ, Scotland

**Keywords:** Obesity, Adolescent, Health-related quality of life

## Abstract

**Background:**

Obesity impairs health related quality of life (HRQL) in adolescents, but most evidence in this area has mostly come from western societies. We wanted to test the hypothesis that obesity impairs HRQL in Kuwaiti adolescents, and to test for differences in HRQL assessed by self-report and parent-proxy report.

**Methods:**

In 500 Kuwaiti 10–14 year olds HRQL was assessed using the Peds QL^TM^ with both adolescent self-reports (n = 500) and parent-proxy reports (n = 374).

**Results:**

Obesity was not significantly associated with HRQL in regression analysis. In a paired comparison of 98 pairs of obese adolescents vs. 98 healthy weight peers, impairment of HRQL reached significance only for physical score (95% CI = −1.5, -9.4), not for psychosocial score or total score. In a paired comparison of parent-proxy vs. self-reports for the obese adolescents, total score (95% CI = −4.9, -10.9), physical score (95% CI = −3.2, -11.0), and psychosocial score (95% CI = −4.2, -10.8) were all significantly lower in the parent reports.

**Conclusions:**

Obesity is not associated with marked impairment of HRQL in adolescents in Kuwait, in contrast to studies in western societies. This may reflect cultural differences in attitudes towards obesity.

## Background

It is now well established, from systematic review and meta-analysis, that obesity impairs health-related quality of life (HRQL) of children and adolescents from western societies [1,2]. There is much less evidence on the extent to which obesity might impair HRQL in adolescents from non-western societies [3-7], but obesity-associated impairment of HRQL has emerged in children, adolescents, and young adults in Malaysia, Taiwan, and Lebanon [3,5-7].

It has been suggested that the impact of child or adolescent obesity on HRQL is influenced by culture [3], but since the evidence base on impairment of HRQL in adolescents is still very limited in geographical scope the hypothesis that culture influences the obesity associated impairment of child or adolescent HRQL has not been tested. For example, it is not clear whether obesity impairs the HRQL of adolescents in the Arabian Gulf States. Deficits in HRQL may drive healthcare utilization by creating a demand for obesity treatment [8-10], and understanding the extent of these deficits in non-western societies is important. One complication is differences in perceptions of HRQL between adolescents and their parents [2,3,10]. The literature suggests that the older the child, the larger the level of disagreement between the self-report and proxy-report of HRQL [11,12]. A full understanding of the impact of obesity on HRQL therefore requires that both the parent proxy-reported and adolescent- reported HRQL are considered [11-17].

The primary purpose of the present study was therefore to test the hypothesis that obesity is associated with impaired HRQL in Kuwaiti adolescents, as in western societies. A secondary aim was to test whether HRQL differed between self-reports and parent-proxy reports for the obese adolescents.

## Methods

### Measurement of HRQL

The Pediatric Quality of Life Inventory (PedsQL™ 4.0, MAPI Research Institute, Lyon, France) was used in the present study, with the Arabic Generic Version. The PedsQL™ is a generic HRQL questionnaire that has both self- and parent-proxy report forms [16]. The forms are available in age-appropriate versions (5-7 years, 8-12 years and 13-18 years), and we used the version appropriate to the age of each study participant in the present study. The PedsQL™ 4.0 is well-established, has been used most commonly in studies of child and adolescent obesity [2], and is a valid and reliable tool which is responsive to clinical change over time [16,17]. The Arabic Generic version of the PedsQL™ used in the present study is valid and reliable, e.g. with internal consistency for the different scales of 0.88-0.92 [18].

The PedsQL™ measures a multidimensional construct that includes 23 items consisting of physical, emotional, social and school performance domains from which a total score, psychosocial score (composite of the emotional function, social function, and school function domains), and physical score are derived. Items are linearly transformed to a 0 to 100 scale, so that the higher the score, the better the HRQL.

### Study participants

The sample was recruited from public (state) schools in Kuwait city, the capital of Kuwait. The original intention was to recruit adolescents from a random sample of public schools, but this proved not to be possible due to limited consent to participate from school head teachers. The Ministry of Education granted approval to the research team to invite all 80 intermediate schools in Kuwait City to participate in the study. Kuwait City has 41 male public intermediate schools and 39 female public intermediate schools. Only 10/80 intermediate school head teachers responded, from 3 girls schools and 7 boys schools, and of these 10, permission to conduct the study at school was granted by only three schools; two male and one female school. Participants and their families provided informed written consent. The PedsQL™4.0 Arabic version was completed independently by the adolescents at school and by their parents at home. The study was approved by the Medical Research Committee of the Ministry of Health and the Ethical Committee of the Ministry of Education in Kuwait.

Study participants were included if within the eligible age range (within grades 5–9, the grade range for intermediate schools, age 10 to 14y), and were either obese or healthy weight as defined below. Overweight pupils were excluded from the study sample in order to provide a marked contrast in weight status between the two groups of interest, and to minimize the impact of any mis-classification arising from use of the body mass index. Other exclusions were based on a brief medical history/checklist aimed at including only apparently healthy adolescents, and excluding participants with serious chronic or acute illness which might affect their HRQL.

#### Assessment of weight status and formation of obese-healthy weight matched pairs

From the three schools which agreed to participate, screening of weight status to determine eligibility was carried out in a total of 1042 pupils (542 boys, 500 girls). Pupils were categorised into healthy weight, overweight, and obese groups relative to reference data from the US CDC 2000 [19]. Obesity was defined as BMI ≥ 95^th^ centile, overweight as BMI of ≥ 85^th^ centile and <95^th^ centile, healthy weight was defined as BMI ≥ 3^rd^ centile and < 85^th^ centile. The US BMI reference data were used in the present study because the absolute BMI values at standard Kuwaiti centiles are extremely high [20], as the Kuwaiti BMI reference was constructed after the obesity epidemic had affected Kuwait [21]. The total number of pupils who did not fulfill the inclusion criteria and/or did not consent was 542, 224 males and 318 females, leaving 500 eligible consenting participants, 318 boys and 182 girls.

A pre-planned paired analysis of HRQL between obese and healthy weight participants, with pair matching for same sex, same school, same school year, and same ethnic group (all participants were Kuwaiti nationals) yielded 98 pairs with 57 paired comparisons in boys and 41 paired comparisons in girls.

#### Statistical analysis

All statistical analyses were performed using Minitab 16.0. Data were checked for normality by descriptive statistics and histograms with normal distribution curves. Both the descriptive data, and differences between groups (e.g. between self versus parent reports) were non-normally distributed and so non-parametric statistical tests were used. For the whole sample self-reports, the skewness and kurtosis values for the physical score were −1.10 and 1.70 , for the psychosocial score these were −1.42 and 2.48 and for the total score they were −1.14 and 1.07 , respectively. For the parent-proxy reports, skewness and kurtosis values for the total score were −0.49 and −0.66, for the psychosocial score −0.44 and −0.51, and for the physical score were −0.84 and −0.30, respectively.

In order to test whether obesity was associated with impaired HRQL two statistical approaches were taken. First, multiple regression was used with HRQL (total score, physical score, and psychosocial score) as the outcome and age, gender, and weight status as the explanatory variables. Second, paired comparisons were undertaken between obese versus healthy weight study participants. Wilcoxon signed-rank tests were used to determine the significance of differences in the paired scores between the obese and the healthy weight groups. We also used Wilcoxon signed-rank tests to determine the significance of any difference in scores between adolescent self-reports and parent-proxy reports in the obese group.

## Results

### Sample characteristics

The final study sample consisted of 500 adolescents, 224 obese and 276 healthy weight, 318 boys (63.6%) and 182 girls (36.4%), median age 12.3y. Of the 500 adolescent participants self- reports were available from all 500, but parent-proxy reports were available from 162 of the obese group and 212 from the healthy-weight group. Characteristics of study participants are summarized in Table 1.

**Table 1:** Characteristics of study participants, median (IQR)

**Variable**	**Healthy weight group**			**Obese group**		
	**Boys**	**Girls**	**Total**	**Boys**	**Girls**	**Total**
	**n = 176**	**n = 100**	**n = 276**	**n = 142**	**n = 82**	**n = 224**
Age	12.3 (2.2)	12.2 (2.3)	12.3 (2.2)	12.6 (1.8)	12.4 (2.1)	12.4 (2.0)
BMI (kg/m^2^)	18.0 (2.9)	18.2 (2.9)	18.1 (3.0)	28.7 (6.8)	29.3 (4.9)	28.8 (5.5)
BMI Z score	0.0 (1.2)	0.1 (1.0)	0.1 (1.1)	2.1 (0.4)	2.0 (0.4)	2.1 (0.4)
HRQL Self-report						
Physical score	96.9 (15.6)	87.5 (19.7)	93.7 (18.8)	90.6 (21.9)	81.3 (28.9)	87.5 (25.0)
Psychosocial score	90.0 (21.6)	86.7 (23.4)	88.3 (20.0)	87.5 (18.4)	82.5 (21.6)	85.0 (18.3)
Total score	92.4 (19.5)	86.4 (20.6)	89.1 (20.3)	88.0 (18.4)	79.3 (21.2)	84.8 (21.2)
HRQL Parent-proxy report	n = 125	n = 87	n = 212	n = 91	n = 71	n = 162
Physical score	90.6 (25.0)	75.0 (43.7)	87.5 (34.4)	84.4 (31.3)	81.3 (43.8)	82.9 (34.4)
Psychosocial score	78.3 (25.0)	70.0 (28.4)	76.7 (26.7)	76.7 (20.0)	80.0 (29.9)	76.7 (25.0)
Total score	82.6 (22.3)	69.6 (31.6)	79.3 (29.3)	76.1 (21.7)	81.5 (29.3)	78.3 (25.3)

#### Test of the hypothesis that HRQL is impaired in the obese group: multiple regression analysis

Of the potential explanatory variables of age, gender, and weight status, only gender had a significant influence on adolescent self-reported HRQL (n = 500; significantly lower total score in girls than boys, p =0.02). In parent-proxy reports, age (lower in older than younger participants, p < 0.01) and gender (lower in girls than boys p < 0.01) had a significant impact on total score. There was no evidence that obesity was associated with impaired HRQL (total score, physical score, psychosocial score) in the regression analyses.

#### Test of the hypothesis that HRQL is impaired in the obese group: paired comparisons of obese versus healthy weight participant

##### Demographic information

Formal matched pairs were selected from the obese and healthy weight groups (n = 98 pairs). Median (IQR) ages of the healthy weight and obese groups were both 12.4 (2.1). Median (IQR) BMI Z scores were 0.1 (1.1) for the healthy weight group and 2.1 (0.4) for the obese group.

##### Self-reports

Summary data are shown in Table 2. There was no significant difference in the total score between groups (n =98 pairs), but the physical score for the healthy weight group was significantly higher than in the obese group.

**Table 2:** Paired comparisons of Health Related Quality of Life (HRQL) for the healthy weight group vs. obese group, median (IQR)

	**Healthy weight group**	**Obese group**	**95% CI**	***P-value***
**Variable**	**n = 98**	**n = 98**		
Self-report	Median (IQR)	Median (IQR)		
Physical score	90.6 (18.7)	87.5 (31.2)	1.5, 9.4	0.007
Psychosocial score	88.3 (18.3)	85.0 (20.4)	−1.6, 7.5	0.224
Total score	88.0 **(**17.7)	84.8 (25.0)	−0.5, 7.1	0.087
Parent-proxy report				
Physical score	86.0 (40.6)	81.3 (31.3)	−4.7, 6.3	0.756
Psychosocial score	76.7 (25.0)	76.7 (28.3)	−3.4, 5.8	0.773
Total score	79.3 (31.5)	78.8 (24.5)	−2.8, 6.0	0.550

Median (IQR) ages of healthy weight and obese groups were both 12.4y (2.1). Median (IQR) BMI Z scores were 0.1 (1.1) for the healthy weight group, and 2.1 (0.4) for the obese group.

##### Parent-proxy reports

As shown in Table 2, there were no significant differences in the paired comparisons of parent-proxy reports between the obese group and healthy weight group.

#### Differences between self-reports and parent-proxy reports for the obese adolescents

There were 162 obese adolescents for the paired comparisons between self-reports and parent-proxy reports (median age = 12.4y, IQR =2.1). Parent-proxy reports were significantly lower than self-reports for physical score (95% CI = 3.2, 11.0), psychosocial score (95% CI = 4.2, 10.8), and total score (95% CI = 4.9, 10.9).

## Discussion

The primary aim of the present study was to test whether the impairment of ‘total’ HRQL associated with obesity from western samples, as measured by the PedsQL™, [1,2] was present in a community sample of adolescents from Kuwait. The results do not suggest that obesity impairs total HRQL markedly in Kuwaiti adolescents*,* in contrast to findings from western samples [1,2] and some non-western samples [3-7]*.*

The present study did find evidence of a deficit in the physical health domain of HRQL , but not for the psychosocial health domain .It is possible that the physical health effects of adolescent obesity might be more universal than psychosocial effects, Psychosocial effects of obesity might be more sensitive to cultural differences, e.g. in the perception of obesity. The apparent lack of impact of obesity on the psychosocial domain of HRQL in the present study might reflect cultural differences in the perception of obesity between Kuwait and western societies, but further research would be required to confirm this. The community-based nature of the sample might also have reduced any potential HRQL deficits associated with obesity. It is possible that HRQL is more greatly impaired in clinic based, treatment-seeking samples [5,6,8,9].

Recent systematic reviews have noted that almost all of the evidence reviewed has been from western countries [1,2]. Very recently, some evidence has begun to emerge from non-western societies [3-7] and this has generally supported the hypothesis that child or adolescent obesity is also associated with reduced HRQL in non-western cultures. The most relevant comparison to the present study is from studies using the PedsQL™ in Arab countries, but such studies are very scarce. Fazah et al. [7], in a community sample of Lebanese 14–18 year olds found that obesity was associated with HRQL impairment, but only in females not males. In contrast, most of the evidence from western societies, as summarized in a recent systematic review [2], suggests that obesity-associated impairment of HRQL applies to both sexes.

We are aware of only one previous study of HRQL in adolescents and young adults in Kuwait: Al-Fayez and Ohaeri [22] used a different instrument to measure HRQL and included older participants than those recruited to the present study, 14-23 y olds. In the study by Al-Fayez and Ohaeri [22] HRQL scores were lower than in samples from western countries, lower in females than males, but the influence of obesity on HRQL was not considered. The reasons why HRQL is lower in girls than boys was not the main focus of the present study, but was consistent with the findings of Al-Fayez and Ohaeri [22].

The secondary aim of the present study was to test the hypothesis that parent-proxy and self- reports of HRQL might differ in obese adolescents. The present study showed that parent-reported HRQL was significantly lower than adolescent-reported HRQL, this emphasizes the potential value of obtaining both parent and self-reports of HRQL [23-25]. Most previous studies have also found that parent-proxy scores for HRQL in obese children and adolescents are lower than scores from self-reports [2]. A detailed discussion of differences in HRQL between parent-proxy versus self reports is beyond the scope of the present manuscript, but a number of detailed studies of the topic have been published [11-15]. The present study simply aimed to establish whether HRQL scores differed between parents and their obese adolescent offspring.

The novelty of the study setting (Arabian Gulf), relatively large sample, heterogeneity of the sample, and ability to compare HRQL between obese versus healthy weight adolescents, were the main study strengths.

The present study also had a number of weaknesses. While the sample size was larger than in many previous studies included in recent systematic reviews [1,2], the school response rate was disappointing and no random selection of schools was possible. In the present study it was not practical to characterize the excluded participants in detail, nor to assess pubertal stage of study participants, yet an assessment of maturation might have added to the information available from chronological age. Most of the psychosocial co-morbidities of child and adolescent obesity tend to worsen with age and/or developmental stage [26]. The present study also focused specifically on HRQL, and cannot address the wider psychosocial co-morbidities of adolescent obesity [1,25-29]. In the present study we were unable to consider all potential influences on HRQL, e.g. we had no measure of socio-economic status (SES) which has a weak association with HRQL in young adults in Kuwait [22], but since the sample was relatively homogenous (same ethnicity, narrow age range, from the same small number of public schools) the range in SES was probably relatively narrow. In our analyses based on paired comparisons of obese and healthy weight adolescents, members of each pair were from the same school year, same sex, and also from the same school. This high degree of pair-wise matching should have minimized the impact of differences other than obesity between the pairs. We were unable to consider any impact of overweight on HRQL, as distinct from obesity, since we excluded the overweight in order to establish adequate contrast between obese and healthy weight groups. However, since the deficits in HRQL associated with obesity in the present study were so small it seems likely that deficits in HRQL associated with overweight might be even smaller. The cross-sectional design of the present study was also a limitation, though most of the research in this area to date has also been cross-sectional [1,2]. Finally, while we did not test the psychometric properties of the PedsQL™ in our sample, in previous studies these have been consistently very positive [7,15-18].

## Conclusion

In conclusion, the present study suggests that adolescent obesity is not associated with marked impairment of HRQL in Kuwait, in contrast to what would have been expected from previous studies of largely western samples. This finding suggests that cultural differences might modify the impact of obesity on HRQL among adolescents.

## Competing interests

The authors have no conflicts of interest to declare.

## Author contributions

Both authors were involved in concept and study design; both drafted the manuscript and revised for critical intellectual content; both gave final approval for submission.

## Pre-publication history

The pre-publication history for this paper can be accessed here:

http://www.biomedcentral.com/1471-2431/13/105/prepub
